# The Long Non-coding RNA ZFAS1 Sponges miR-193a-3p to Modulate Hepatoblastoma Growth by Targeting RALY via HGF/c-Met Pathway

**DOI:** 10.3389/fcell.2019.00271

**Published:** 2019-11-08

**Authors:** Xichun Cui, Zhifang Wang, Liwen Liu, Xin Liu, Dandan Zhang, Jianhao Li, Jianming Zhu, Juntao Pan, Da Zhang, Guangying Cui

**Affiliations:** ^1^Department of Pediatric Surgery, The First Affiliated Hospital of Zhengzhou University, Zhengzhou, China; ^2^Department of Endocrinology, The First Affiliated Hospital of Zhengzhou University, Zhengzhou, China; ^3^Precision Medicine Center, The First Affiliated Hospital of Zhengzhou University, Zhengzhou, China; ^4^Department of Pathology, The First Affiliated Hospital of Zhengzhou University, Zhengzhou, China

**Keywords:** ZFAS1, miR-193a-3p, RALY, HGF, c-Met, hepatoblastoma

## Abstract

Hepatoblastoma (HB) is the most common and aggressive malignant hepatic neoplasm in childhood and the therapeutic outcomes remain undesirable due to its recurrence and metastasis. Recently, long non-coding RNA (lncRNA) zinc finger antisense 1 (ZFAS1) has been reported to be an oncogenic gene in multiple cancers. However, the expression status and specific role of ZFAS1 involved in cancer progression of human HB remain unknown. This study aimed to identify the role of ZFAS1/miR-193a-3p/RALY axis in the development of HB. Here we showed that the expression of ZFAS1 was significantly upregulated in both HB tissues and cell lines. High ZFAS1 expression was significantly associated with aggressive tumor phenotypes and poorer overall survival in HB. *In vitro* and *in vivo* function assays indicated that silencing of ZFAS1 significantly suppressed HB cell proliferation and invasion. Furthermore, miR-193a-3p was identified to be the target of ZFAS1. Subsequently, RALY was confirmed to be regulated by miR-193a-3p/ZFAS1 axis. Mechanistically, our results indicated that the ZFAS1 participated to the progression of HB via regulating the HGF/c-Met signaling. Collectively, these data demonstrated that ZFAS1 acted as an oncogene to promote initiation and progression of HB by regulating miR-193a-3p/RALY (RALY Heterogeneous Nuclear Ribonucleoprotein) axis via HGF/c-Met Pathway, which provides an efficient marker and new therapeutic target for HB.

## Introduction

Hepatoblastoma (HB) is one of the most common and highly invasive primary malignant liver tumor in pediatric patients, which accounts for approximately 50% of pediatric liver cancers, occurring mostly within the first 2 years of life (Kremer et al., 2014). Over the last three decades, despite regional and local control of HB have improved owing to the application of adjuvant chemotherapy, surgical resection and liver transplantation, the prognosis for patients in advanced HB stages remains very poor, little is still known about its pathogenesis (Cristobal et al., 2019). Thus, it is particularly important to identify valid biomarkers for early diagnosis and treatment of HB.

Long non-coding RNAs (lncRNAs) are a class of RNAs with longer than 200 nucleotides, lacking a complete open reading frame (ORF). Recent studies have demonstrated that lncRNAs are aberrantly expressed in various human cancers (Guo et al., 2019; Li et al., 2019; Zhang and Fan, 2019), including HB (Dong et al., 2017). Simultaneously, accumulating evidences have shown that specific lncRNAs act as tumor suppressor genes or oncogenes via mediating tumor progression. Long non-coding RNA-CRNDE (Dong et al., 2017), TUG1 (Dong et al., 2016) and OIP5-AS1 (Zhang et al., 2018) promote oncogenesis in HB, while experimental verification shows that LINC01314 acts as a tumor suppressor in HB (Lv et al., 2018). ZFAS1 is one of the recognized lncRNAs, which represents a snoRNA host gene locating on chromosome 20q13.13. Dysregulation of ZFAS1 has been reported in hepatocellular carcinoma (Li T. et al., 2015), breast cancer (Hansji et al., 2016) and many other types of human cancers (Xu et al., 2018; He et al., 2019). However, there is not known reports about the clinical significance and underlying mechanism of ZFAS1 in the progression of HB.

MicroRNAs (miRNAs) are endogenous non-coding RNAs, with ∼22 nucleotides, which can bind to the 3′ untranslated region (3′UTR) of target mRNA to regulate gene expression, leading to the degradation of the mRNA or translational inhibition of functional proteins. An increasing number of evidences had revealed that miRNAs were extensively involved in numerous pathological processes, including diabetes mellitus (Ghai et al., 2019), atherosclerosis (Bruen et al., 2019), and tumors. Meanwhile, there are many reports of miRNAs related to HB, such as miR-17 (Ecevit et al., 2019), miR-21 (Liu et al., 2016), and miR-492 (Von Frowein et al., 2011). Currently, miR-193a-3p has been reported to act as tumor suppressor in many cancers. For instance, hepatocellular carcinoma (Qian et al., 2017), bladder cancer (Li Y. et al., 2015) and colorectal cancer (Pekow et al., 2017). However, little is known about the expression status and specific role of miR-193a-3p in HB development.

Notably, promising evidence has shown that lncRNAs, including ZFAS1, are validated to act as competing endogenous RNAs (ceRNAs) to regulate gene expression by competitively binding to miRNAs (Li et al., 2017, 2018). However, whether ZFAS1 affects the biological behavior of HB cells by regulating miR-193a-3p has not been determined. Therefore, exploration of the functional role and underlying molecular mechanism of lncRNA–miRNA–mRNA crosstalk may become a key development in HB.

In the present study, we validated an interaction between ZFAS1 and miR-193a-3p which modulated HB cell proliferation and invasion by targeting RALY. Based on the findings, a novel regulatory pathway composed of ZFAS1/miR-193a-3p/RALY was provided for HB diagnosis and therapy.

## Materials and Methods

### Dataset Acquisition

Two independent microarrays, including GSE75271 and GSE75283 databases, were extracted from the Gene Expression Omnibus (GEO).^1^ Their characteristics, such as cohort ID, RNA-Seq platform, number of samples, publication year and country, are showed in Supplementary Table S1. DESeq package in R language was carried out to compare the lncRNA expression data from HB tissues and non-tumor samples in GEO dataset. A heatmap was created by using the pheatmap package in R language.

### Patients and Specimens

Seventy paired HB tissues and adjacent non-tumor samples were obtained from the First Affiliated Hospital of Zhengzhou University (Zhengzhou, China) between April 2011 and December 2018 (ZZU cohort). The follow-up and clinicopathological data were listed in Table 1. All patients did not receive radiotherapy or chemotherapy before the surgery. All samples were frozen in liquid nitrogen immediately after surgery and then stored at −80°C until the total RNA extraction.

**TABLE 1:** Association of ZFAS1 expression and clinicopathological features.

**Clinicopathological features**		**No. of cases (%)**	**ZFAS1**		***P*-value**
			**Low**	**High**	
			**(*n* = 28)**	**(*n* = 42)**	
Age (years)	≤Median	35	15	20	0.626
	>Median	35	13	22	
Gender	Male	44	17	27	0.762
	Female	26	11	15	
Tumor size	≤10 cm	36	13	23	0.494
	>10 cm	34	15	19	
Vascular invasion	Absent	55	24	31	0.234
	Present	15	4	11	
AFP	≤100 ng/ml	36	15	21	0.769
	>100 ng/ml	34	13	21	
Histologic type	Epitheliated	52	30	22	0.330
	Mixed	18	8	10	
Metastasis	Absent	52	26	26	**0.003**
	Present	18	2	16	
Recurrence	Absent	57	26	31	**0.044**
	Present	13	2	11	
COG stage	Stage I-II	41	23	18	**0.001**
	Stage III-IV	29	5	24	

*Bold values indicate statistical significance, *P* < 0.05.*

### Cell Culture

The HB cell lines (HepG2 and HuH-6), normal liver cell lines (Chang liver and L02) and embryonic kidney cell lines (HEK293) used in this study were purchased from the Chinese Academy of Sciences (Shanghai, China). Cells were cultured in Dulbecco’s modified Eagle’s medium (DMEM) supplemented with 10% fetal bovine serum and 100 U/ml penicillin/streptomycin (Corning, NY, United States) in a humid incubator (5% carbon dioxide, 95% air) at 37°C. The detail of these cells was shown in the Supplementary Table S2.

### RNA Extraction and Quantitative RT-PCR (qRT-PCR)

Total RNA was extracted utilizing Trizol reagent (Life Technologies, Garlsbad, CA, United States). TransScript First- Strand cDNA Synthesis SuperMix (TransGen, Beijing, China) was used to generate cDNA. qPCR reactions were performed using PowerUp SYBR Green kit (ABI, Foster City, CA, United States) and QuantStudio 6 System (ABI, Foster City, CA, United States). Data was analyzed using the comparative Ct method (2−^ΔΔ^Ct). β-actin served as the internal control.

### Cell Transfection Assays

ZFAS1 pcDNA3.1 vector (ZFAS1), RALY pcDNA3.1 vector (RALY) and empty vector (vector) were subcloned into the vector pcDNA3.1 (Invitrogen, Carlsbad, CA, United States). miR-193a-3p mimic, negative control oligonucleotides (mimic-NC), miR-193a-3p inhibitor, negative control oligonucleotide (NC inhibitor), small interfering RNA of ZFAS1 or RALY (si-ZFAS1, si-RALY) and scramble siRNA of ZFAS1 or RALY (siSCR) were purchased from RiboBio (Guangzhou, China). Cells were transfected using lipofectamine 2000 (Thermo Fisher, CA, United States) following to the manufacturer’s protocols. qRT-PCR was performed at 48–72 h later to determine the transfection efficiency. For further *in vivo* experiments, sh-ZFAS1 and sh-SCR were obtained from RiboBio (Guangzhou, China) and constructed into HB cell lines.

### Luciferase Assays

Using Lipofectamine 2000 (Invitrogen Co, Carlsbad, CA, United States) following the manufacturer’s procedures, HEK293 cells were co-transfected pcDNA3.1 ZFAS1-wt, pcDNA3.1 ZFAS1-mut, pcDNA3.1 RALY-wt or pcDNA3.1 RALY-mut together with miR-193a-3p mimic or the control. After 48 hours, the Dual-Luciferase Reporter Assay System (Promega, Madison, WI, United States) was used to determine the luciferase activity.

### RIP Assay

The RNA immunoprecipitation (RIP assay) was performed using a Thermo Fisher RIP kit (Thermo Fisher Scientific, Waltham, MA, United States). According to the manufacturer’s protocol, cells were collected and lysed in RIP lysis buffer, the lysates were conjugated with human anti-Argonaute2 (Ago2) antibody (Millipore, Billerica, MA, United States) or negative control normal mouse anti-IgG (Millipore, Billerica, MA, United States) in magnetic beads. Subsequently, the retrieved RNA was assayed by qRT-PCR.

### Cell Proliferation and Colony Formation Assays

Cell growth was evaluated according to the protocol using CCK-8 Kit (Beyotime, China). The DNA synthesis rate was evaluated through Edu assay kit (Ribobio, Guangzhou, China). For colony formation assays, transfected cells were placed in 6-well plates and incubated for 2 weeks, then, all cells were fixed, stained, photographed and analyzed.

### Cell Migration and Invasion Assays

Wound-healing assay was performed to measure the cell migration ability. HepG2 and HuH-6 cells (5 × 10^6^) were seeded into six-well plates. A 1 mm wide wound was created using a 200-μL sterile tip after 90% confluence was reached. The wounded areas were observed and photographed every 24 h under microscope.

Cell invasion assay was conducted utilizing transwell chambers coated with matrigel (BD, Franklin Lakes, NJ, United States). Upper chambers were seeded at 5 × 10^4^ transfected cells using serum-free media, and the lower chambers were filled with DMEM with 10% FBS. Cell invasive capacity was assessed by counting invading cells under a microscope (40 × 10). Each chamber was measured for five random fields of view.

### Tumor Xenografts

Mice assays were approved by the Animal Health Committee of Zhengzhou University. The male nude mice (4–6 weeks) were purchased from Beijing Vital River Laboratory (Beijing, China). HuH6 cell lines transfected with ZFAS1 knockdown lentivirus (sh-ZFAS1) and empty lentivirus control (sh-SCR) were subcutaneously implanted into the lower flank of nude mice. Tumor growth was examined every week. Mice were euthanized at 5 weeks post implantation. Photographs were taken using the IVIS Lumina II system. The tumor tissues were weighed and extracted for further IHC staining.

### Immunohistochemical Staining

Immunohistochemical (IHC) staining was performed according to our previous study (Sun et al., 2016), which was performed using the avidin-biotin immunoperoxidase technique. Sections (4-μm-thick) were immunostained as previously described.

### Western Blot

RIPA buffer was utilized to extract total protein from HB cells. Following extraction, BCA assays (Beyotime, Shanghai, China) were performed to quantify all proteins. 20 μg protein samples were loaded onto 12% SDS-PAGE. Following separation, the samples were transferred from the gel to the nitrocellulose membranes (Millipore, Burlington, MA, United States) and blocked with 5% BSA/PBST for 1 h. The membranes were then incubated with anti-β-actin, RALY or indicated antibodies at 4°C(C overnight. After washed with PBST, the secondary antibody incubation was performed for 2 h and the membranes were exposed with the photographic film for visualization. Additional Supplementary Table S3 listed the information of antibodies.

### Statistical Analysis

GraphPad Prism software (version 7.0, United States) and SPSS (Version 23.0, IBM, Seattle, WA, United States) were carried out for statistical analyses. Differences between two groups were analyzed by Student’s *t*-test or the Mann–Whitney *U* test. Clinicopathological characteristics in HB were analyzed by chi-square tests. Overall survival (OS) was calculated with Kaplan- Meier curves and log-rank tests. Cox regression in univariate and multivariate analyses were performed to verify the independent prognostic factors. Correlation was performed by Spearman rank analysis. A *p*-value < 0.05 was considered to be statistically significant. All data were presented as the mean ± standard deviation (SD).

## Results

### Overexpression of ZFAS1 Correlates With Clinicopathological Features and Poor Progression in HB

**FIGURE 1:**
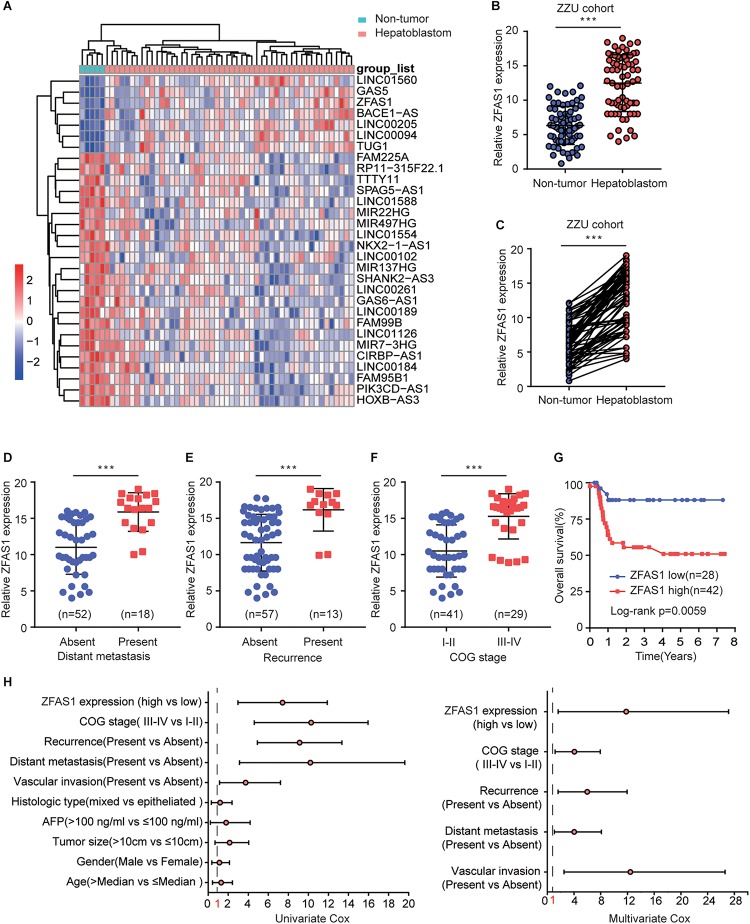
Overexpression of ZFAS1 correlates with clinicopathological features and poor prognosis in HB patients. **(A)** Top 30 differentially expressed lncRNAs based on GEO database (GSE75271). **(B)** Quantitative qRT-PCR analysis of ZFAS1 expression in 70 human HB tissues and the paired adjacent normal tissues. **(C)** ZFAS1 expression levels in HB tissues compared with their adjacent non-tumor tissues. The correlation between the expression levels of ZFAS1 with tumor distant metastasis **(D)**, tumor recurrence **(E)** and COG stage **(F)**. **(G)** Kaplan-Meier survival analysis of overall survival rate between HB patients with low or high ZFAS1 expression. **(H)** Forest plot depicting the result of univariate and multivariate Cox regression analysis. ^∗∗∗^*p* < 0.001.

We first assessed the GEO database (GSE75271). Subsequently, we conducted the literature research. ZFAS1 was finally selected for in-depth investigation due to its unknown biological possesses in HB and significantly differential expression in GEO database (Supplementary Figure S1A). A heatmap showed top30 selected lncRNAs, including 7 up-regulated genes and 23 down-regulated genes (Figure 1A). To draw a comprehensive conclusion, we further examined the expression of ZFAS1 in 70 pairs of matched HB and normal tissues by qRT-PCR using the ZZU cohort. Consistent with our observation, ZFAS1 was significantly up-regulated in HB tissues (Figures 1B,C). Moreover, overexpression of ZFAS1 significantly correlated with aggressive clinic phenotypes (Figures 1D–F and Table 1) and poorer overall survival data (Figure 1G). More interestingly, univariate and multivariate Cox regression analysis revealed that high ZFAS1 expression was a potent independent risk indicator for survival in HB patients (Figure 1H and Table 2). Overall, these data showed that ZFAS1 upregulation might play an essential role in the progression of HB.

**TABLE 2:** Univariate and multivariate analyses of overall survival of hepatoblastoma.

**Clinicopathological features**		**Univariate analyses**		***P*-value**	**Multivariate analyses**		***P*-value**
		**HR**	**95% (CI)**		**HR**	**95% (CI)**	
Age (years)	<Median	1.027	0.436–2.420	0.951			
	>Median						
Gender	Male	0.882	0.365–2.130	0.781			
	Female						
Tumor size	≤10 cm	1.674	0.693–4.044	0.252			
	>10 cm						
AFP	≤100 ng/ml	0.972	0.225–4.192	0.969			
	>100 ng/ml						
Histologic type	Epitheliated type	0.868	0.317–2.378	0.783			
	Mixed						
Vascular invasion	Absent	2.863	1.136–7.217	**0.026**	1.000		
	Present				8.158	2.500–26.617	**0.001**
metastasis	Absent	7.841	3.133–19.623	**0.000**	1.000		
	Present				2.940	1.068–8.091	**0.037**
Recurrence	Absent	7.171	2.989–17.204	**0.000**	1.000		
	Present				4.388	1.612–11.946	**0.004**
COG stage	Stage I-II	2.699	1.103–6.457	**0.003**	1.000		
	Stage III-IV				3.064	1.182–7.946	**0.021**
ZFAS1	Low	4.746	1.397–16.128	**0.013**	1.000		
	High				6.657	1.632–27.148	**0.008**

*Bold values indicate statistical significance, *P* < 0.05.*

### Knockdown of ZFAS1 Suppresses HB Cell Proliferation and Invasion Capacity *in vitro*

**FIGURE 2:**
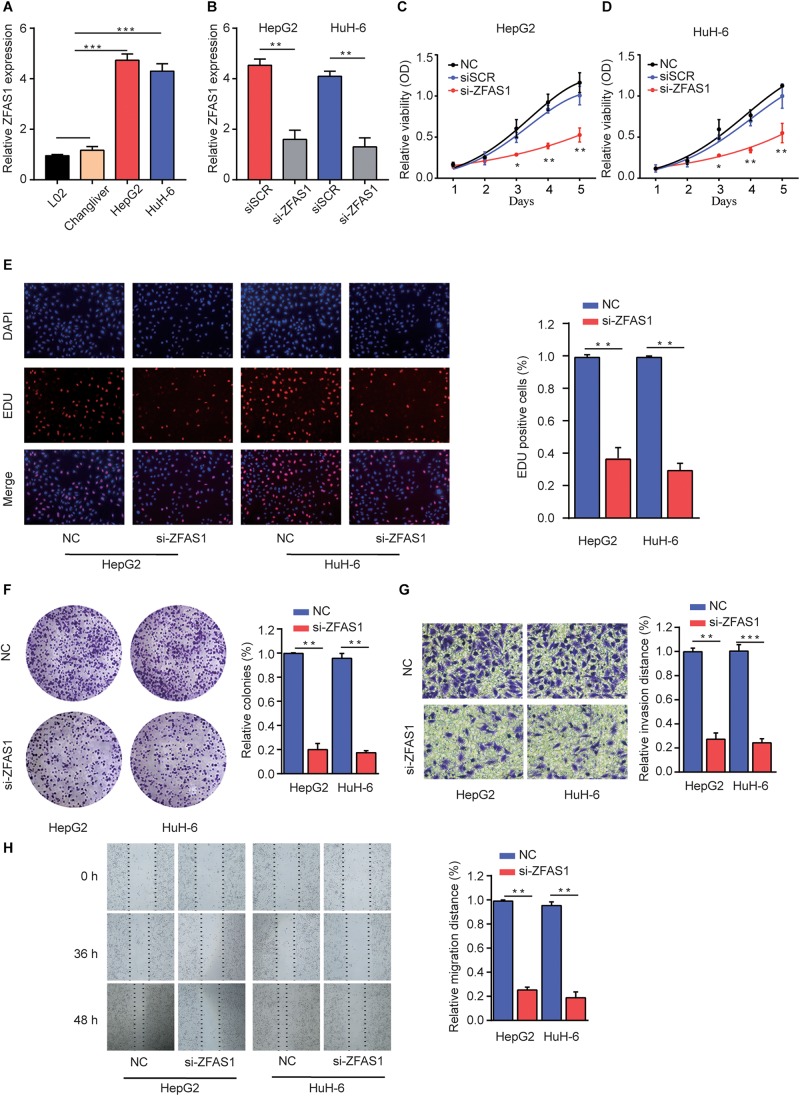
Knockdown of ZFAS1 suppresses HB cell proliferation, migration and invasion. **(A)** qRT-PCR analysis of ZFAS1 expression in normal liver cell lines (L02 and Chang Liver) and HB cell lines (HepG2 and HuH-6). **(B)** qRT-PCR analysis of ZFAS1 expression in HepG2 or HuH-6 cells transfected with siSCR or si-ZFAS1. Cell proliferation capacity was analyzed by CCK-8 assay **(C,D)**, EDU staining assay **(E)** and colony formation assay **(F)**. **(G)** The invasion capability of HepG2 or HuH-6 cells transfected with NC or si-ZFAS1 was analyzed by transwell assay. Transwell assays were repeated for three times and relative quantification analysis was based on grayscale values. **(H)** The migration capability of HepG2 or HuH-6 cells transfected with NC or si-ZFAS1 was analyzed by wound-healing assay at indicated time points. ^∗^*p* < 0.05, ^∗∗^*p* < 0.01, ^∗∗∗^*p* < 0.001.

To explore the regulatory effects of ZFAS1 in HB cells, we investigated the ZFAS1 expression in normal liver cells (L02 and ChangLiver) and HB cell lines (HepG2 and HuH-6). qRT-PCR result showed that the expression levels of ZFAS1 were much higher in HepG2 and HuH-6 cells than that in normal liver cells (Figure 2A). Next, we employed siRNA to knockdown the ZFAS1 expression in HepG2 and HuH-6 cells (Figure 2B and Supplementary Figure S1E). CCK-8 proliferation assay (Figures 2C,D), EDU staining assay (Figure 2E) and colony formation assay (Figure 2F) demonstrated that the cell proliferation ability was dramatically suppressed after silencing ZFAS1. In addition, the migratory and invasive ability were also remarkably suppressed in HepG2 or HuH-6 cells transfected with si-ZFAS1 using transwell (Figure 2G) and wound-healing assays (Figure 2H). Simultaneously, we demonstrated that ZFAS1 overexpression promoted HB cell proliferation and invasion *in vitro* (Supplementary Figure S2). These results exhibited the potential carcinogenicity of ZFAS1 in HB.

### Knockdown of ZFAS1 Inhibits Tumor Formation Rate *in vivo*

**FIGURE 3:**
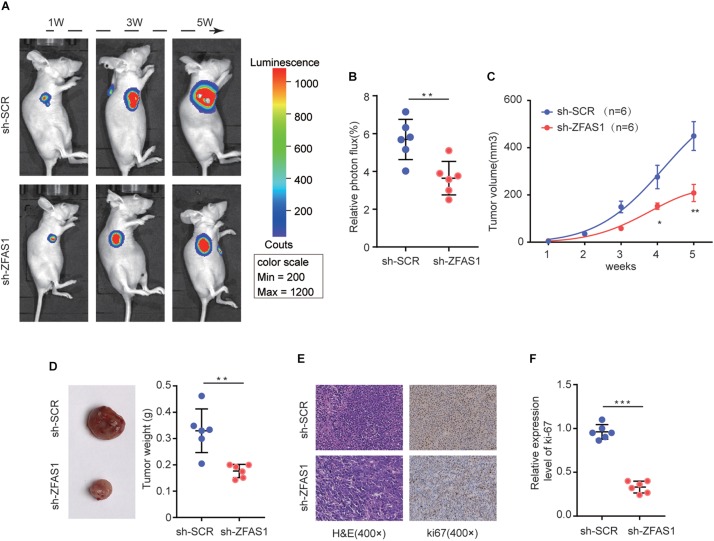
The effect of ZFAS1 on tumor growth *in vivo.* **(A)** Representative image of Luciferase signal by a live imaging system. **(B)** Relative photon flux in sh-ZFAS1 group and sh-SCR group were quantified and analyzed using the IVIS imaging system. **(C,D)** Quantitative analysis of xenografted tumor volume and weight. **(E,F)** Representative hematoxylin and eosin (H&E) staining images and relative expression levels of immunohistochemical staining (IHC) for ki-67 in tumor sections from sh-ZFAS1 group and sh-SCR group. ^∗^*p* < 0.05, ^∗∗^*p* < 0.01, ^∗∗∗^*p* < 0.001.

To verify the function of ZFAS1 on HB tumorigenesis *in vivo*, HuH-6 cells with stable ZFAS1 knockdown (transfected with sh-ZFAS1) or corresponding control (transfected with sh-SCR) were implanted into nude mice (BALB/c) to construct a xenograft model. Knockdown of ZFAS1 significantly inhibited tumor growth as shown by luciferase photon flux and tumor volume at different time points after implantation (Figures 3A–C). Consistently, the tumors in sh-ZFAS1 group had markedly lower tumor weight compared with those in sh-SCR group (Figure 3D). Additionally, we further examined the proliferation marker Ki67 expression and found its expression was also decreased in the sh-ZFAS1 group by IHC analysis (Figures 3E,F). These results further indicate the oncogenic ability of ZFAS1 in HB.

### ZFAS1 Binds to miR-193a-3p and Negatively Regulates Its Expression

**FIGURE 4:**
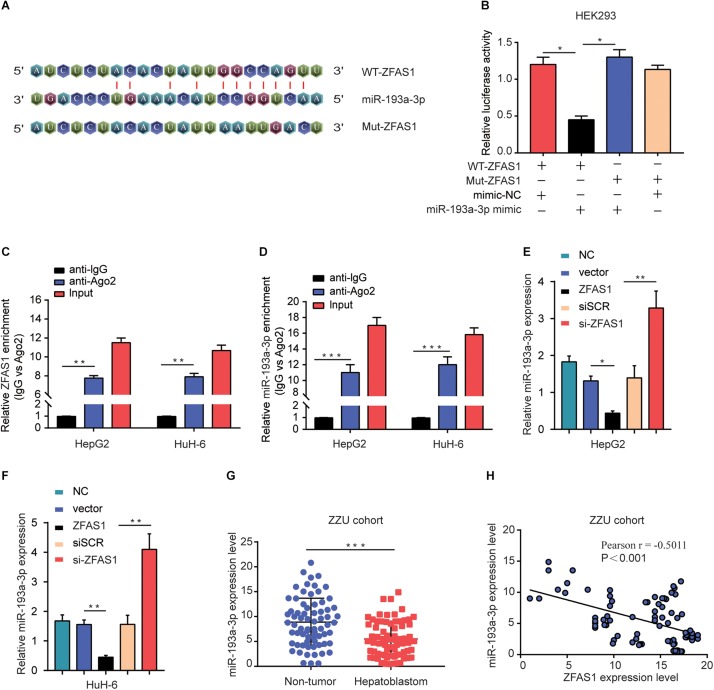
ZFAS1 negatively regulates miR-193a-3p expression. **(A)** Graphical representation of the potential binding sites between ZFAS1 and miR-193a-3p. **(B)** The relative luciferase activity of pZFAS1-miR-193a-3p-wt and pZFAS1-miR-193a-3p-mut was tested. **(C,D)** RIP assay was performed to determine the association between ZFAS1 and miR-193a-3p in HepG2 cells. **(E)** miR-193a-3p expression level in HepG2 cells transfected with ZFAS1 or si-ZFAS1 was shown. **(F)** miR-193a-3p expression level in HuH-6 cells transfected with ZFAS1 or si-ZFAS1 was shown. **(G)** miR-193a-3p expression in HB tissues were quantified by qRT- PCR analysis. **(H)** The negative correlation between ZFAS1 and miR-193a-3p was displayed by Pearson’s correlation curve. ^∗^*p* < 0.05, ^∗∗^*p* < 0.01, ^∗∗∗^*p* < 0.001.

Recently, accumulating evidence has suggested that lncRNAs have been acknowledged as ceRNAs competitively share miRNAs recognition sites with mRNAs to modulate cancer-related gene expression. To explore the target miRNAs of ZFAS1 in HB, an online bioinformatics software (Starbase v3.0) was used to predict the potential targets for ZFAS1. miR-193a-3p was predicted to have putative ZFAS1 binding sites (Figure 4A). The results of Dual-luciferase reporter assay showed that luciferase activity of pZFAS1-miR-193a-3p-wt was significantly decreased, which validated the binding of miR-193a-3p with ZFAS1 (Figure 4B). In addition, the RIP assay revealed that both ZFAS1 and miR-193a-3p were enriched in Ago2 containing beads (Figures 4C,D). To further investigate the regulatory impact between ZFAS1 and miR-193a-3p, HepG2 and HuH-6 cells were transfected with pcDNA-ZFAS1, si-ZFAS1 or corresponding controls. It was observed that knockdown of ZFAS1 significantly increased miR-193a-3p expression and miR-193a-3p was significantly downregulated in pcDNA-ZFAS1 transfected HepG2 and HuH-6 cells (Figures 4E,F). In addition, the qRT-PCR data showed that miR-193a-3p was downregulated and negatively correlated with ZFAS1 in HB tissues (Figures 4G,H). In GEO dataset (GSE75283), the miR-193a-3p expression levels were also dramatically downregulated in HB tissues compared with normal tissues (Supplementary Figure S1B). These data suggested that ZFAS1 could directly target miR-193a-3p and negatively regulate its expression.

### Knockdown of ZFAS1 Suppresses HB Cell Proliferation and Invasion by Targeting miR-193a-3p

**FIGURE 5:**
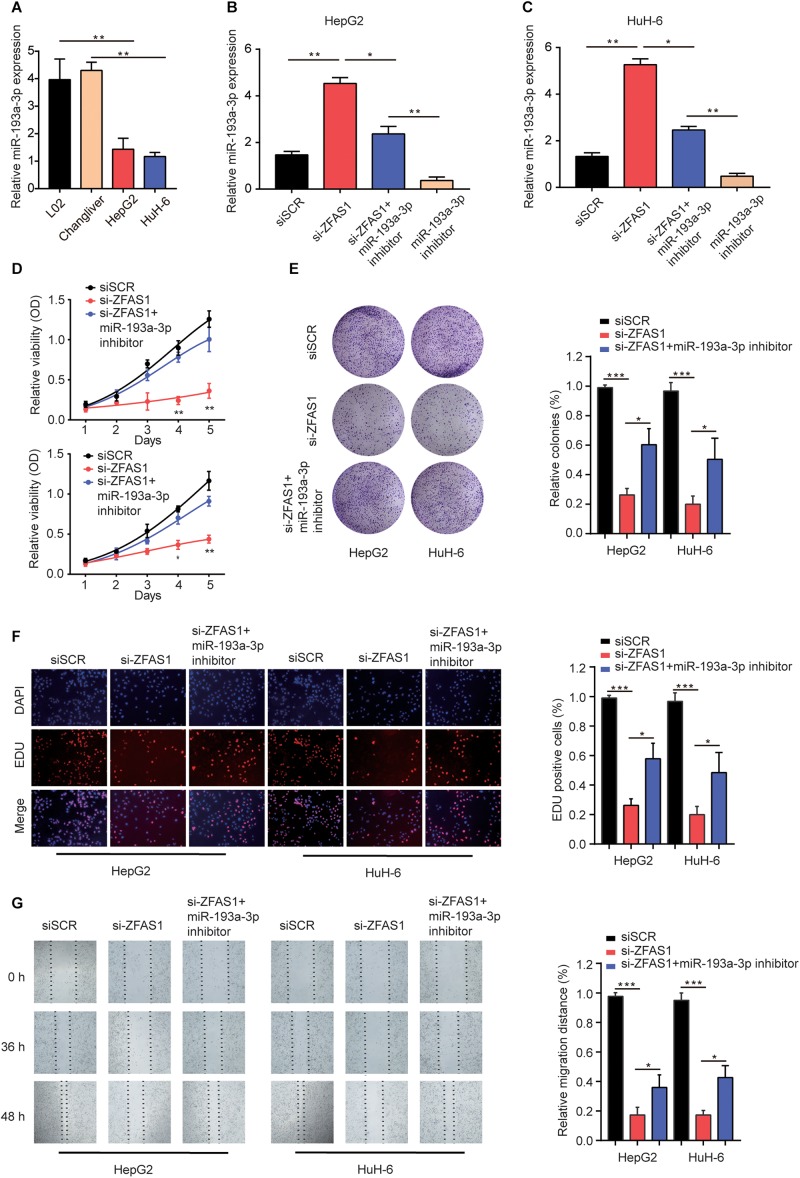
Knockdown of ZFAS1 suppresses HB cell proliferation and invasion by targeting miR-193a-3p. **(A)** qRT-PCR analysis of miR-193a-3p expression in normal liver cell lines (L02 and Chang Liver) and HB cell lines (HepG2 and HuH-6). **(B,C)** HepG2 or HuH-6 cells were transfected with siSCR, si-ZFAS1 or si-ZFAS1 and miR-193a-3p inhibitor. The expression levels of DPEP1 in different groups were analyzed by qRT-PCR. Cell proliferation activity of HepG2 or HuH-6 cells was assessed by CCK-8 assay **(D)**, Cell colony formation assay **(E)** and EDU staining assay **(F)**. **(G)** Cell migration ability of HepG2 or HuH-6 cells in different groups was analyzed by wound healing assay. ^∗^*p* < 0.05, ^∗∗^*p* < 0.01, ^∗∗∗^ *p* < 0.001.

To demonstrate the effects of ZFAS1/miR-193a-3p axis on HB cells, we investigated the miR-193a-3p expression in normal liver cells (L02 and ChangLiver) and HB cell lines (HepG2 and HuH-6). qRT-PCR results showed that the expression levels of miR-193a-3p were much lower in HepG2 and HuH-6 cells than that in normal liver cells (Figure 5A). Next, we transfected HepG2 or HuH-6 cells with siSCR, si-ZFAS1 or si-ZFAS1 and miR-193a-3p inhibitor. qRT-PCR was performed to determine the transfection efficiency (Figures 5B,C). The results of CCK-8 assay and colony formation assay showed that the downregulation of miR-193a-3p restored the suppressive effect of si-ZFAS1 on cell proliferation capacity (Figures 5D,E). EDU staining assay presented a similar conclusion (Figure 5F). Meanwhile, wound healing assay revealed that knockdown of miR-193a-3p partially reversed the suppressive effect induced by si-ZFAS1 on cell migration ability (Figure 5G). In conclusion, all these data indicate that downregulation of ZFAS1 inhibits HB cell mobility by targeting miR-193a-3p.

### RALY Is a Direct Target of miR-193a-3p, ZFAS1 Regulates RALY Expression by Sponging miR-193a-3p

**FIGURE 6:**
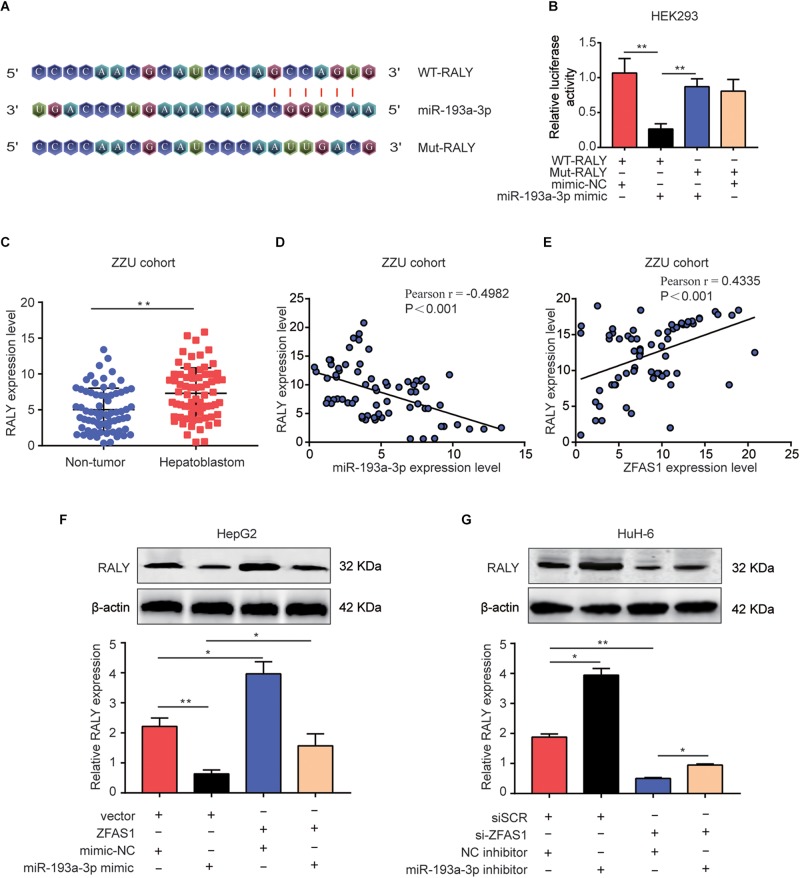
ZFAS1 regulates RALY expression by sponging miR-193a-3p. **(A)** Graphical representation of the potential binding sites between miR-193a-3p and RALY. **(B)** The relative luciferase activity was tested after co-transfection with RALY wide type, RALY mutant type and miR-193a-3p mimic. **(C)** qRT-PCR analysis of RALY expression in 70 paired human HB tissues and the adjacent normal tissues. **(D)** Correlation between RALY and miR-193a-3p was measured by Pearson’s correlation curve. **(E)** Correlation between RALY and ZFAS1 was measured by Pearson’s correlation curve. **(F)** The effects of ZFAS1, miR-193a-3p mimic, ZFAS1 & miR-193a-3p mimic on mRNA and protein levels of RALY. **(G)** The effects of si-ZFAS1, miR-193a-3p inhibitor, si-ZFAS1 & miR-193a-3p inhibitor on mRNA and protein levels of RALY. Western blot assays were repeated for three times. ^∗^*p* < 0.05, ^∗∗^*p* < 0.01.

Lately, there had been mounting reports about the ceRNA hypothesis, which revealed lncRNAs could involve in lncRNA–miRNA–mRNA crosstalk and ceRNA networks. First, we initially verified RALY as potential target of miR-193a-3p using online bioinformatics software (Starbase v3.0 and Target-Scan)^2^ (Figure 6A). Next, Dual-luciferase reporter assay was performed to validate the binding of miR-193a-3p with RALY (Figure 6B). In addition, the mRNA expression levels of RALY in 70 HB tissues were up-regulated compared with the paired non-tumor tissues (Figure 6C). Then, Pearson analysis showed a negative correlation between RALY and miR-193a-3p in HB samples (Figure 6D). On the contrary, RALY was positively associated with ZFAS1 expression levels in HB tissues (Figure 6E). Consistent results were observed in GEO dataset (GSE75271) (Supplementary Figures S1C,D). To demonstrate if ZFAS1 and RALY compete for the binding of miR-193a-3p, RALY expression was analyzed. Interestingly, the mRNA and protein levels of RALY were significantly increased in ZFAS1 overexpression group but were downregulated when transfected with miR-193a-3p mimic. Co-transfected with ZFAS1 and miR-193a-3p mimic could reverse the regulatory effects of ZFAS1 on RALY expression (Figure 6F). Moreover, ZFAS1 downregulation significantly inhibited RALY expression, while the expression level of RALY could be reversed by co-transfecting si-ZFAS1 and miR-193a-3p inhibitor (Figure 6G).

**FIGURE 7:**
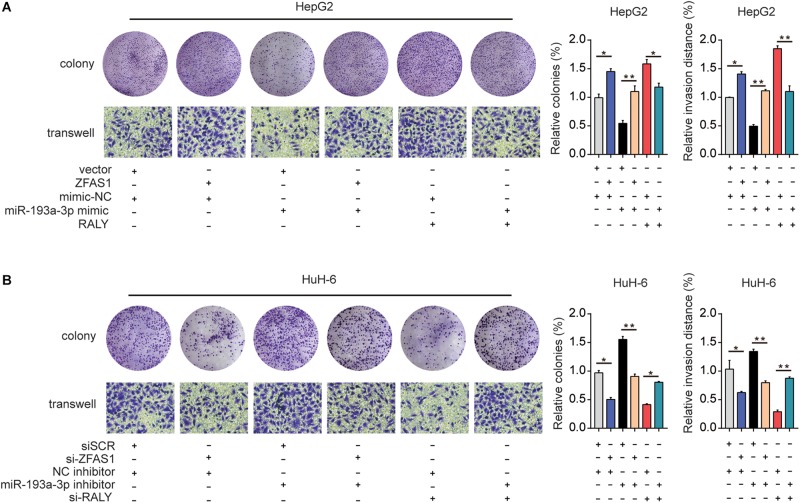
The effect of ZFAS1/miR-193a-3p/RALY axis on HB cells biological behavior *in vitro*. **(A)** ZFAS1, RALY and miR-193a-3p mimic were co-transfected into HeG2 cells, Cell proliferation and invasion ability were assessed by colony formation assay and transwell assay. **(B)** si-ZFAS1, si-RALY and miR-193a-3p inhibitor were co-transfected into HuH-6 cells, cell proliferation and invasion ability were assessed by colony formation assay and transwell assay. ^∗^*p* < 0.05, ^∗∗^*p* < 0.01.

Finally, ZFAS1, RALY and miR-193a-3p mimic were co-transfected into HeG2 cells, the proliferation and invasion capacity were investigated (Figure 7A). Additionally, downregulation of ZFAS1 or RALY expression attenuated the proliferation and invasion in HuH-6 cells. Knockdown of miR-193a-3p reversed the regulatory effects of ZFAS1 or RALY (Figure 7B). Collectively, these observations suggest that ZFAS1 regulate RALY in the carcinogenesis of HB by acting as a molecular sponge to modulate miR-193a-3p.

### ZFAS1 Regulates HGF/c-Met Signaling in Human HB

**FIGURE 8:**
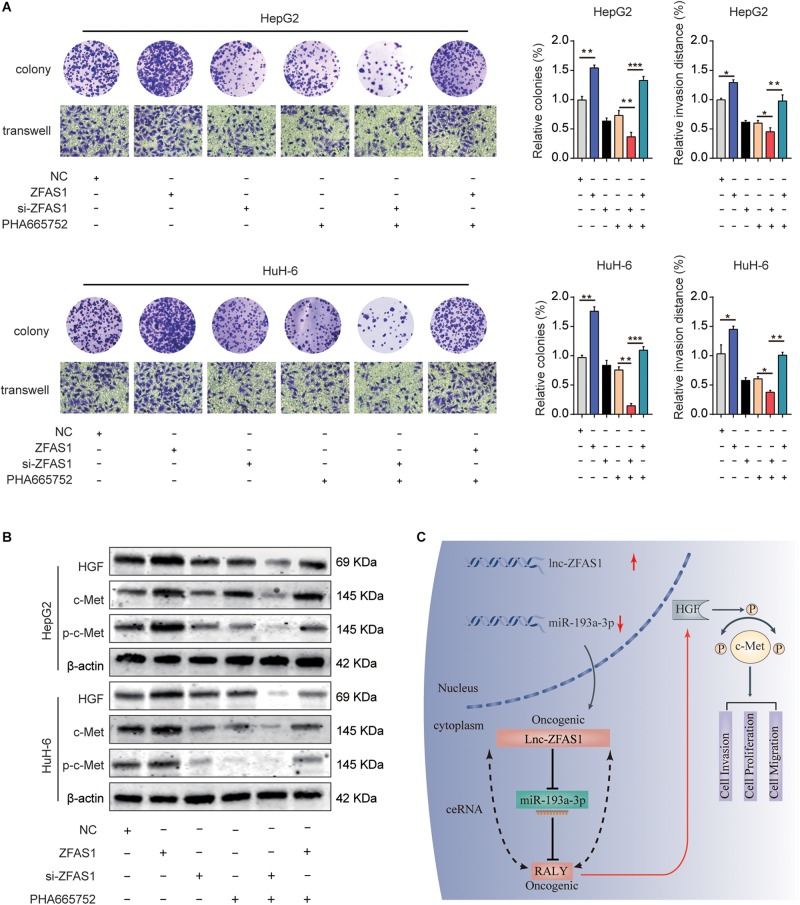
ZFAS1 regulates HGF/c-Met signaling in human HB. **(A)** ZFAS1, si-ZFAS1 and PHA665752 were co-transfected into HeG2 and HuH-6 cells, cell proliferation and invasion ability were assessed by colony formation assay and transwell assay. **(B)** Expression levels of HGF, c-Met and p-c-Met in HepG2 and HuH-6 transfected with ZFAS1, si-ZFAS1 and PHA665752 were analyzed by western blot. **(C)** A mechanism diagram depicting that the ZFAS1/miR-193a-3p/RALY axis affects the progression of HB through the HGF/c-Met signaling. ^∗^*p* < 0.05, ^∗∗^*p* < 0.01, ^∗∗∗^*p* < 0.001.

To explore the potential function mechanism of ZFAS1 in HB, *in vitro* function assays were performed. PHA665752, a selective small-molecule c-Met inhibitor, potently counteracted the effect of ZFAS1 in promoting cell proliferation and invasion (Figure 8A). In addition, we found that the protein expression levels of HGF, c-Met and p-c-Met were significantly decreased in ZFAS1 knockdown group and increased in ZFAS1 overexpression group. Simultaneously, ZFAS1 downregulation could further enhance the inhibitory effect of PHA665752 while ZFAS1 overexpression could reverse the inhibitory effect of PHA665752 in HepG2 and HuH-6 cells (Figure 8B). To validate our results, we repeated the western blot experiments using the miR-193a-3p inhibitor in ZFAS1-knockdown cells. Consistently, we found that the protein expression levels of HGF, c-Met and p-c-Met (Tyr1234/1235) were significantly decreased in WT-ZFAS1 knockdown group. In addition, miR-193a-3p inhibitor could reverse the inhibitory effect of ZFAS1 downregulation (Supplementary Figure S3).

## Discussion

Emerging evidences had shown that dysregulation of lncRNAs could play an essential role in human malignant tumors, including HB. For example, long non-coding RNA-TUG1 correlated with tumor angiogenesis and progression in HB (Dong et al., 2016). Dong et al. (2017) showed that RNA-CRNDE might act as an functional target and unpleasant prognostic factor for HB. Zhang et al. (2018) also found that high expression of OIP5-AS1 promoted HB cells proliferation and invasion. ZFAS1, a novel biomarker of lncRNA, has been reported to be up-regulated and plays an important role in tumor progression. Ectopic overexpression of ZFAS1 promoted metastasis in hepatocellular carcinoma (Li T. et al., 2015). Liu et al. (2017) also demonstrated that ZFAS1 might be served as a potential biomarker of Chinese solid cancer patients. Consistent with these reports, in our present study, we showed that ZFAS1 was significantly upregulated in HB tissues and cell lines. Additionally, high expression of ZFAS1 was positively associated with distant metastasis, recurrence, advanced COG stage and unpleasant prognosis. Moreover, ZFAS1 knockdown impeded the mobility of HB cells *in vitro*, while dampened tumor growth *in vivo.* These results elucidated the potential carcinogenicity of ZFAS1 in HB.

Previous studies showed that lncRNAs could serve as natural miRNA sponges or ceRNAs to regulate specific miRNAs. For instance, lncRNAs CCAT1 (Deng et al., 2015), XIST (Chen et al., 2016) and LINC00152 (Cai et al., 2017) were reported to promote cancer progression by functioning as miRNA sponges in hepatocellular carcinoma, gastric cancer and gallbladder cancer. In the present study, bioinformatics prediction displayed that miR-193a-3p, which had been confirmed to be suppressor in diverse human cancers, including hepatocellular carcinoma (Qian et al., 2017), lung cancer (Fan et al., 2017) as well as gastric cancer (Fan et al., 2017), might have potential ZFAS1 binding sites. Simultaneously, luciferase reporter and RIP assays validated an endogenous interaction between ZFAS1 and miR-193a-3p. Furthermore, we observed a negative correlation between ZFAS1 and miR-193a-3p. These data demonstrated that ZFAS1 might act as a molecular sponge of miR-193a-3p in HB, which agreed well with the results of previous studies. Such as, ZFAS1 sponges miR-484 to promote cell proliferation and invasion in colorectal cancer (Xie et al., 2018). Additionally, lncRNAs ZFAS1 sponges miR-486 to promote osteosarcoma cells metastasis and progression (Li et al., 2017).

RALY is known to be a member of the heterogeneous nuclear ribonucleoprotein (hnRNP) gene family. The oncogenic role and regulatory effects have been reported in human cancers. Recent study indicated that overexpression of RALY predicted poor prognosis and acted as an oncogene in hepatocellular carcinoma (Zhu et al., 2018). In addition, RALY acted as a RNA binding protein to modulate metastatic potential of breast cancer cells (Bondy-Chorney et al., 2017). Consistent with these findings, our study also uncovered that RALY expression was significantly up-regulated in HB tissues compared with adjacent normal tissues. Meanwhile, we confirmed miR-193a-3p had a potential target site in the 3′-UTR of RALY. Pearson analysis showed a negative correlation between RALY and miR-193a-3p in HB samples. On the contrary, RALY was positively associated with ZFAS1 expression levels in HB tissues. In addition, our study showed that ZFAS1 could positively adjust RALY expression by sponging for miR-193a-3p. More importantly, co-transfection assay demonstrated that altered levels of ZFAS1, miR-193a-3p and RALY were associated with progression of HB cells. Taken together, these data predicted that ZFAS1 involved in ceRNA networks and ZFAS1-miR-193a-3p-RALY crosstalk could modulate HB progression. However, due to the complexity of tumor microenvironment, it is possible that many other mechanisms may involve in regulating miR-193a-3p expression, such as methylation or RNA decay/stability. Further exploration for these mechanisms will be needed.

Emerging evidences have shown that the overexpression and hyper-activation of c-Met when it binds to its ligand HGF, plays a pivotal role in tumorigenesis, including HB. For instance, HGF/c-Met related activation of β-catenin may be a treatment option of HB (Purcell et al., 2011). Ongoing efforts have identified an important role of HGF/c-Met signaling in the promotion of tumor angiogenesis, growth and metastasis of HB (Goyal et al., 2013). In our study, overexpression of ZFAS1 was positively correlated with the expression levels of HGF/c-Met signaling-associated molecules, while ZFAS1 knockdown suppressed the activation of the HGF/c-Met signaling. Moreover, ZFAS1 overexpression could reverse the inhibitory effect of PHA665752. Overall, our results provide solid evidences that ZFAS1 is involved in HB progression through controlling the HGF/c-Met signaling.

In summary, our study showed that ZFAS1 served as a ceRNA to regulate RALY by sponging miR-193a-3p and played an oncogenic role during HB progression via HGF/c-Met Pathway (Figure 8C). This study displayed a possible molecular mechanism of tumorigenesis and progression in HB involving ZFAS1/miR-193a-3p/RALY axis, which could be acted as an intriguing biomarker and potential therapeutic target for HB.

## Data Availability Statement

All data generated or analyzed during this study are included either in this article/Supplementary Material.

## Ethics Statement

The study methodology conformed to the standard set by the Declaration of Helsinki and was approved by the First Affiliated Hospital of Zhengzhou University (Henan, China). All patients had signed inform consent forms.

## Author Contributions

XC, ZW, and DZ performed all the experimental work. LL, JL, and DDZ participated in data analysis. JZ, XL, JL, and DDZ designed and conducted the animal experiment. GC, JP, and DZ conceived and participated in the design of the study. XC wrote the manuscript. All authors read and approved the final manuscript.

## Conflict of Interest

The authors declare that the research was conducted in the absence of any commercial or financial relationships that could be construed as a potential conflict of interest.

## Footnotes

1 https://www.ncbi.nlm.nih.gov/geo/

2 microRNA.org

## Supplementary Material

FIGURE S1 The expression of ZFAS1/miR-193a-3p/RALY axis in GEO dataset. **(A)** The ZFAS1 expression levels in HB tissues compared with normal tissues in GSE75271 dataset. **(B)** The miR-193a-3p expression levels in HB tissues compared with normal tissues in GSE75283 dataset. **(C)** The RALY expression levels in HB tissues compared with normal tissues in GSE75271 dataset. **(D)** Correlation between RALY and ZFAS1 was measured by Pearson’s correlation curve in GSE75271 dataset. **(E)** We repeated the qRT-PCR experiments to explore the transfection efficiency of ZFAS1 siRNA in HuH-6 cells for 5 days. After 5 days qRT-PCR result showed that the expression of ZFAS1 was still at lower levels. ^∗^*p* < 0.05, ^∗∗^*p* < 0.01, ^∗∗∗^*p* < 0.001.

FIGURE S2 Up-regulation of ZFAS1 promotes HB cell proliferation and invasion. **(A,B)** Cell proliferation capacity was analyzed by CCK-8 assay and colony formation assay **(C)**. **(D)** The invasion capability of HepG2 or HuH-6 cells transfected with NC or ZFAS1 was analyzed by transwell assay. ^∗^*p* < 0.05, ^∗∗^*p* < 0.01.

FIGURE S3 ZFAS1 regulates HGF/c-Met signaling via ZFAS1/miR-193a-3p/RALY axis in human HB. Expression levels of HGF, c-Met and p-c-Met in HepG2 and HuH-6 transfected with NC, si-WT-ZFAS1, si-Mut-ZFAS1 and miR-193a-3p inhibitor were analyzed by western blo.

TABLE S1 GEO information used in this study.

TABLE S2 Cell lines used in this study.

TABLE S3 Information on antibodies used in this study.

## Abbreviations

c-Met: cell surface-mesenchymal-epithelial transition
GEO: Gene Expression Omnibus
HB: hepatoblastoma
HGF: hepatocyte growth factor
IHC: immunohistochemistry
lncRNA: long non-coding RNA
qRT-PCR: quantitative real-time PCR
RALY: RBP-associated with lethal yellow mutation
ZFAS1: zinc finger antisense 1.
