# Supplementary figures and images for: The Long Non-coding RNA ZFAS1 Sponges miR-193a-3p to Modulate Hepatoblastoma Growth by Targeting RALY via HGF/c-Met Pathway
Source: Front Cell Dev Biol. 2019 Nov 8;7:271. doi: 10.3389/fcell.2019.00271 (PMC6856658; doi:10.3389/fcell.2019.00271)

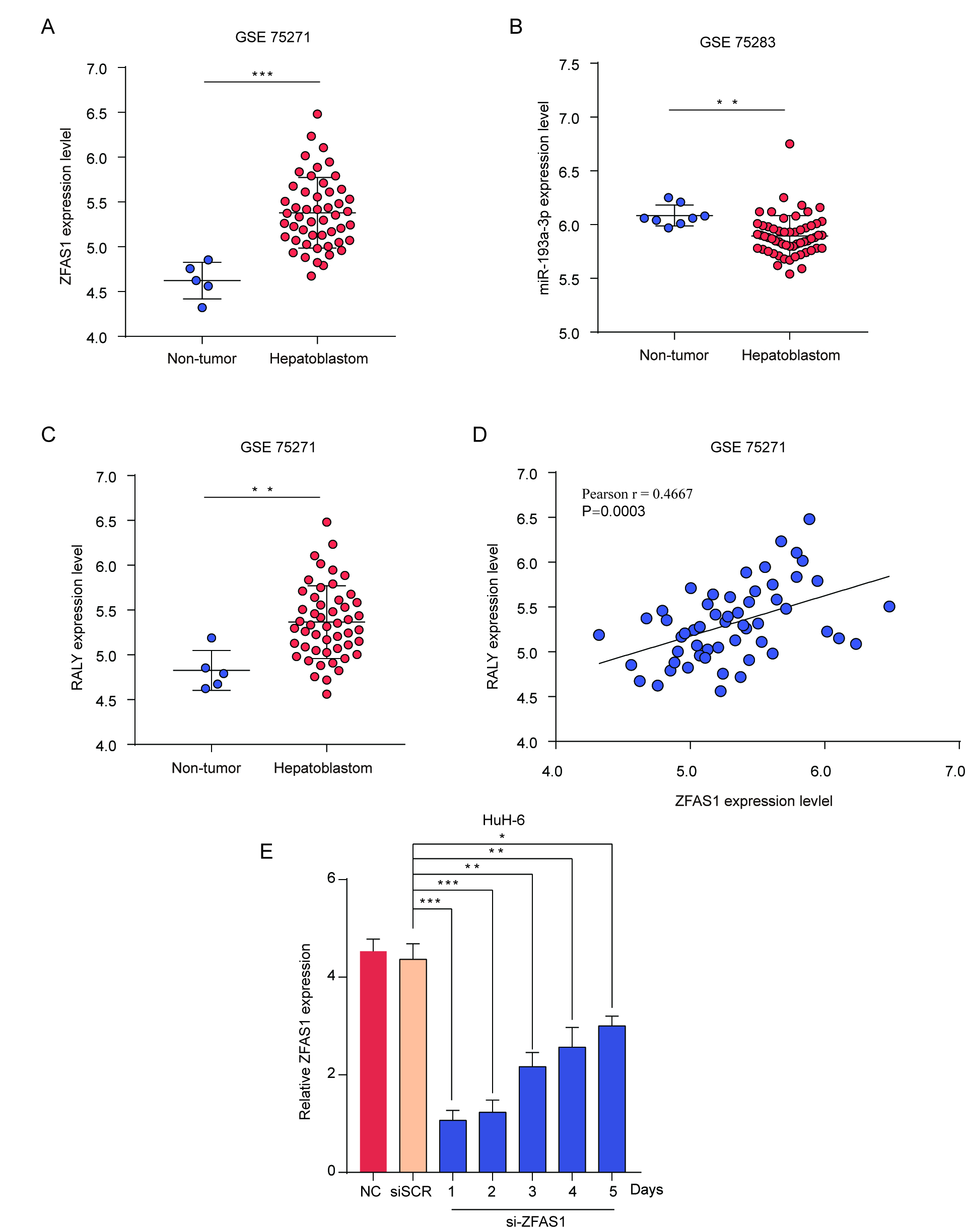

Supplement: FIGURE S1 — The expression of ZFAS1/miR-193a-3p/RALY axis in GEO dataset. (A) The ZFAS1 expression levels in HB tissues compared with normal tissues in GSE75271 dataset. (B) The miR-193a-3p expression levels in HB tissues compared with normal tissues in GSE75283 dataset. (C) The RALY expression levels in HB tissues compared with normal tissues in GSE75271 dataset. (D) Correlation between RALY and ZFAS1 was measured by Pearson’s correlation curve in GSE75271 dataset. (E) We repeated the qRT-PCR experiments to explore the transfection efficiency of ZFAS1 siRNA in HuH-6 cells for 5 days. After 5 days qRT-PCR result showed that the expression of ZFAS1 was still at lower levels. ∗p < 0.05, ∗∗p < 0.01, ∗∗∗p < 0.001. [file Image_1.TIF]

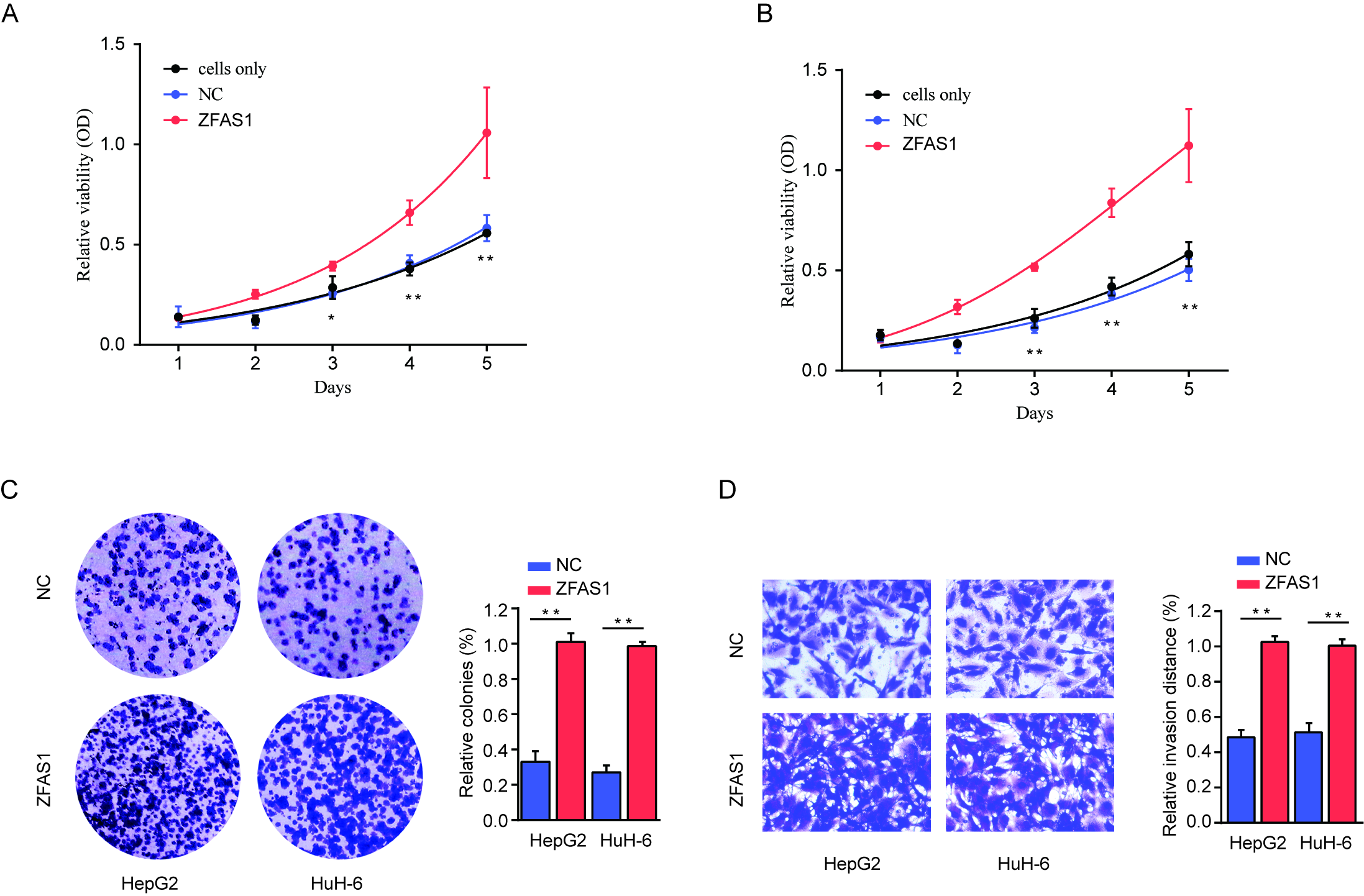

Supplement: FIGURE S2 — Up-regulation of ZFAS1 promotes HB cell proliferation and invasion. (A,B) Cell proliferation capacity was analyzed by CCK-8 assay and colony formation assay (C). (D) The invasion capability of HepG2 or HuH-6 cells transfected with NC or ZFAS1 was analyzed by transwell assay. ∗p < 0.05, ∗∗p < 0.01. [file Image_2.TIF]

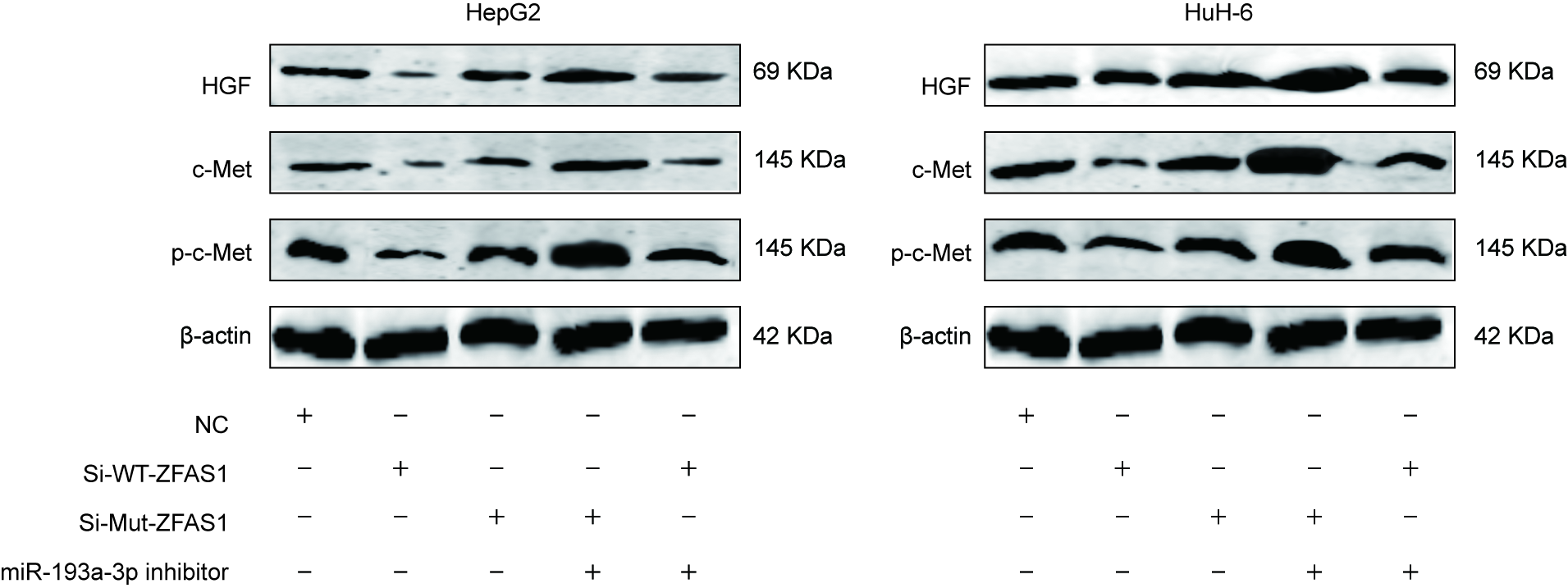

Supplement: FIGURE S3 — ZFAS1 regulates HGF/c-Met signaling via ZFAS1/miR-193a-3p/RALY axis in human HB. Expression levels of HGF, c-Met and p-c-Met in HepG2 and HuH-6 transfected with NC, si-WT-ZFAS1, si-Mut-ZFAS1 and miR-193a-3p inhibitor were analyzed by western blo. [file Image_3.TIF]
