# Supplementary material for: The Long Non-coding RNA ZFAS1 Sponges miR-193a-3p to Modulate Hepatoblastoma Growth by Targeting RALY via HGF/c-Met Pathway
Source: Front Cell Dev Biol. 2019 Nov 8;7:271. doi: 10.3389/fcell.2019.00271 (PMC6856658; doi:10.3389/fcell.2019.00271)
Supplement: TABLE S1 — GEO information used in this study. [file Table_1.DOCX]

**Table S1. GEO information used in this study**

| GEO ID | Platforms | Non-tumor | Tumor | Year | Country |
| --- | --- | --- | --- | --- | --- |
| GSE75271 | Affymetrix mRNA microarray | 5 | 50 | 2016 | USA |
| GSE75283 | Agilent miRNA microarray | 8 | 57 | 2017 | USA |
| Total |  | **13** | **107** |  |  |

**Table S2. Cell lines used in this study**

| **Cell lines** | **Cell type** | **Source** | **Country** |
| --- | --- | --- | --- |
| HepG2 | Hepatoblastoma cell | Cell Bank of the Chinese  Academy of Science | China |
| HuH-6 | Hepatoblastoma cell | Cell Bank of the Chinese  Academy of Science | China |
| Chang liver | Normal liver cell | Cell Bank of the Chinese  Academy of Science | China |
| L02 | Normal liver cell | Cell Bank of the Chinese  Academy of Science | China |
| HEK293 | Embryonic kidney cell | Cell Bank of the Chinese  Academy of Science | China |

**Table S3. Information on antibodies used in this study**

| **Antibody** | **WB** | **IHC** | **Specificity** | **Company** |
| --- | --- | --- | --- | --- |
| β-actin | 1:5000 | / | Mouse monoclonal | Proteintech Group, China |
| RALY | 1:500 | / | Rabbit Polyclonal | Proteintech Group, China |
| Ki-67 | / | 1:500 | Rabbit Polyclonal | Proteintech Group, China |
| HGF | 1:500 |  | Rabbit Polyclonal | Proteintech Group, China |
| c-Met | 1:200 |  | Rabbit Polyclonal | Proteintech Group, China |
| p-c-Met | 1:1000 |  | Rabbit monoclonal | Cell Signaling Technology, USA |
